# The impact of COVID-19 on clinical research for Neglected Tropical Diseases (NTDs): A case study of bubonic plague

**DOI:** 10.1371/journal.pntd.0010064

**Published:** 2021-12-20

**Authors:** Tsinjo Fehizoro Rasoanaivo, Josephine Bourner, Ravaka Niaina Randriamparany, Théodora Mayouya Gamana, Voahangy Andrianaivoarimanana, Mily Harijaona Raherivelo, Harivelo Randriamampionona, Minoarisoa Rajerison, Mihaja Raberahona, Alex Paddy Salam, Tansy Edwards, Piero L. Olliaro, Rindra Vatosoa Randremanana

**Affiliations:** 1 Institut Pasteur de Madagascar, Antananarivo, Madagascar; 2 Centre for Tropical Medicine and Global Health, Nuffield Department of Medicine, University of Oxford, Oxford, United Kingdom; 3 Service de Santé de District Ambositra, Ambositra, Madagascar; 4 Service de Santé de District Manandriana, Manandriana, Madagascar; 5 Centre Infectiologie Charles Mérieux, Université d’Antananarivo, Antananarivo, Madagascar; 6 Service des Maladies Infectieuses, Centre Hospitalier Joseph Raseta Befelatanana, Antananarivo, Madagascar; 7 London School of Hygiene and Tropical Medicine, London, United Kingdom; University of Texas Medical Branch at Galveston, UNITED STATES

## Abstract

**Background:**

Among the many collaterals of the COVID-19 pandemic is the disruption of health services and vital clinical research. COVID-19 has magnified the challenges faced in research and threatens to slow research for urgently needed therapeutics for Neglected Tropical Diseases (NTDs) and diseases affecting the most vulnerable populations. Here we explore the impact of the pandemic on a clinical trial for plague therapeutics and strategies that have been considered to ensure research efforts continue.

**Methods:**

To understand the impact of the COVID-19 pandemic on the trial accrual rate, we documented changes in patterns of all-cause consultations that took place before and during the pandemic at health centres in two districts of the Amoron’I Mania region of Madagascar where the trial is underway. We also considered trends in plague reporting and other external factors that may have contributed to slow recruitment.

**Results:**

During the pandemic, we found a 27% decrease in consultations at the referral hospital, compared to an 11% increase at peripheral health centres, as well as an overall drop during the months of lockdown. We also found a nation-wide trend towards reduced number of reported plague cases.

**Discussion:**

COVID-19 outbreaks are unlikely to dissipate in the near future. Declining NTD case numbers recorded during the pandemic period should not be viewed in isolation or taken as a marker of things to come. It is vitally important that researchers are prepared for a rebound in cases and, most importantly, that research continues to avoid NTDs becoming even more neglected.

## Author summary

The COVID-19 pandemic has forced health systems to re-evaluate service provision, leading to the diversion of resources and widespread disruption of research activities. In particular, the pandemic has jeopardised the progress of research for Neglected Tropical Diseases (NTDs), which has historically faced considerable challenges.

Many NTDs suffer a lack of robust clinical evidence to support current treatment recommendations and previous attempts to conduct clinical trials have been hampered by field conditions.

This article presents the case of a randomised controlled trial for an NTD that was initiated at the start of the pandemic and explores the factors that may have influenced enrolment. Importantly, this article highlights mitigation strategies that have been considered by the trial team to ensure that trial activities can be maintained.

The reduction in reporting of NTDs during the pandemic does not necessarily mean that there are fewer cases at the community level or that this trend will continue. As outbreaks of COVID-19 persist, strategies to mitigate the effect of continued disruption are critical to ensure that, even with a downward trend in NTD case notification, studies addressing an urgent research need can continue and NTDs don’t become even more neglected than they already are.

## Introduction

As a result of the relentless spread of the SARS-Cov2 virus [1], two years into the COVID-19 pandemic, governments continue to implement a number of preventative measures to curb transmission and prevent health systems from collapsing under the influx of severely ill patients. With vaccines still largely unavailable outside high-income countries, these measures include border closures, restrictions on domestic travel, enforcement of minimum business activities, closure of non-essential workspaces, social distancing, restrictions on gatherings, among many others. [2,3]

The impact of these measures and the continued increase of COVID-19 transmission have forced health systems to re-evaluate the services they can provide with the resources available, which has led to the prioritisation of some services over others. Research activities have been widely disrupted as a result. While these effects are seen almost everywhere in the world, low- and middle-income countries are hit hardest.

Clinical research studies for diseases other than COVID-19 have experienced marked declines in patient recruitment, increases in protocol deviations, and modifications to trial conduct and methodology which may affect trial objectives. [4–6] These challenges are magnified in clinical research of neglected and re-emerging infectious diseases, which can be episodic, low-incidence, highly influenced by climate and have historically been hampered by challenging field conditions and notorious lack of funding. These characteristics are also shared with the priority diseases identified by the World Health Organization research and development (WHO R&D) Blueprint that represent a significant public health threat—the definition of which encompasses epidemic-prone diseases which lack (sufficient) countermeasures. [7] While COVID-19 has highlighted the need to be prepared for these diseases, research studies of epidemic-prone diseases at large have been considerably hampered throughout the pandemic.

In this article we explore the effects of the COVID-19 pandemic on the IMASOY (Isika Mitsabo tArimo ho an’ny SOa Iombonana/*Together let us treat the bubo for the common good*) trial [8]—a randomised controlled trial assessing a ciprofloxacin monotherapy treatment regimen against the current standard of care treatment—an aminoglycoside plus ciprofloxacin—for bubonic plague in Madagascar. The IMASOY trial has a target sample size of 190 confirmed and probable cases of plague to be recruited over three transmission seasons (August—March) in the districts that have historically reported the highest case load. The trial began in February 2020—two weeks after the WHO declared COVID-19 a public health emergency of international concern.

Plague is an epidemic-prone disease caused by the bacterium, *Yersinia Pestis*, with a high mortality rate of around 20–25% [9] and, in its pneumonic form, is considered a High Consequence Infectious Disease. [10,11] The reported global burden of disease for plague is low (243 cases were reported in 2018) and the majority of cases lie in low-income countries (98% of global cases are currently reported in Madagascar and the Democratic Republic of Congo alone) with sporadic cases reported in a few middle- and high-income countries, such as the United States of America and China. [11] However, larger outbreaks of plague have been reported in recent years: in 2017, 418 probable and confirmed cases of pneumonic plague were reported during a large outbreak in Madagascar alongside 139 probable and confirmed bubonic cases. [9] Like many other neglected and re-emerging infections, such as Ebola virus disease, plague is highly stigmatised, which can complicate case detection and treatment, as well as transmission and control. [12]

As is the case for other similarly neglected infectious diseases of poverty, there is no available plague vaccine and treatment regimens currently recommended under national and international guidelines are not supported by solid evidence from large-scale, good-quality trial data. Streptomycin has been widely used historically as the first-line treatment recommendation for plague in many countries. Several *Y*. *Pestis* strains have however demonstrated antimicrobial resistance to streptomycin [13] and its limited global supply is anticipated to become completely unavailable within the next 1–2 years. There is therefore an urgent need to assess available therapeutics for the treatment of plague.

Here, we present the impact of the COVID-19 pandemic on the IMASOY trial and discuss the wider public health implications of the pandemic on plague. This article highlights the need for researchers, funders, sponsors, committees and regulatory bodies to review trial recruitment data and national disease surveillance data in the context of the concurrent pandemic—not in isolation. We also present the strategies that the IMASOY trial has implemented and considered to ensure research efforts and results continue to be generated for a disease that has historically been neglected.

## Methods

To assess the impact of COVID-19 on recruitment to the IMASOY trial, we (i) reviewed the number of plague notifications reported to the Central Plague Laboratory (Ministry of Public Health, Madagascar) under the national plague programme, and (ii) collected data from health registries on the number of all-cause consultations that took place at 13 participating *Centres de Santé de Base* (CSB; peripheral health centres) in the districts of Ambositra and Manandriana—with a population of approximately 314 968 and 111 693, respectively [14]—and at the Centre Hospitalier de Référence Régionale (CHRR)—the large regional referral hospital that serves the Amoron’i Mania region, with a population of approximately 837,116 [14]. Data were collected for the period before the start of the pandemic (February 2019 to February 2020) and after the start of the pandemic (February 2020 to February 2021). We also considered the other external factors that may have influenced the reduction in plague notifications during the pandemic and, more broadly, the challenges of conducting the trial that were met by the research team.

### Analysis

All descriptive analyses were conducted using Excel. We present the absolute difference and percentage difference in all-cause consultations in the pre-pandemic (February 2019 to February 2020) and pandemic (February 2020 to February 2021) periods. The percentage difference in all-cause consultations for the CSBs is presented as the monthly median across all 13 health centres from which data was collected.

We also present the number of plague cases notified across three consecutive transmission seasons (August 2018 to April 2019; August 2019 to April 2020; August 2020 to April 2021) alongside the percentage decrease in the number of case notifications year on year, as well as the percentage decrease in case notifications from 2018 to 2021.

Finally, the number of suspected plague cases enrolled in the IMASOY trial in each of the four participating districts is presented as a proportion of the total suspected cases of plague notified to the Ministry of Public Health in Madagascar.

## Results

From March 2020 to April 2021, 175 cases of COVID-19 were confirmed in Ambositra and five cases were confirmed in Manandriana. These case numbers are likely to be an underestimate due to reduced diagnostic capacity in the districts and the syndromic approach to case management taken at the health centres.

### The impact of COVID-19 on health centre attendance

A decrease in the number of all consultations was reported at six (46%) health centres during the pandemic period. Health centre managers attributed this change in caseload to patients’ perception that health centres were a hotspot for transmission and feared contracting COVID-19 if they were to attend. The remaining seven (54%) health centres observed either a small increase or minor fluctuations in consultation numbers. (S1 Fig).

**Fig 1:**
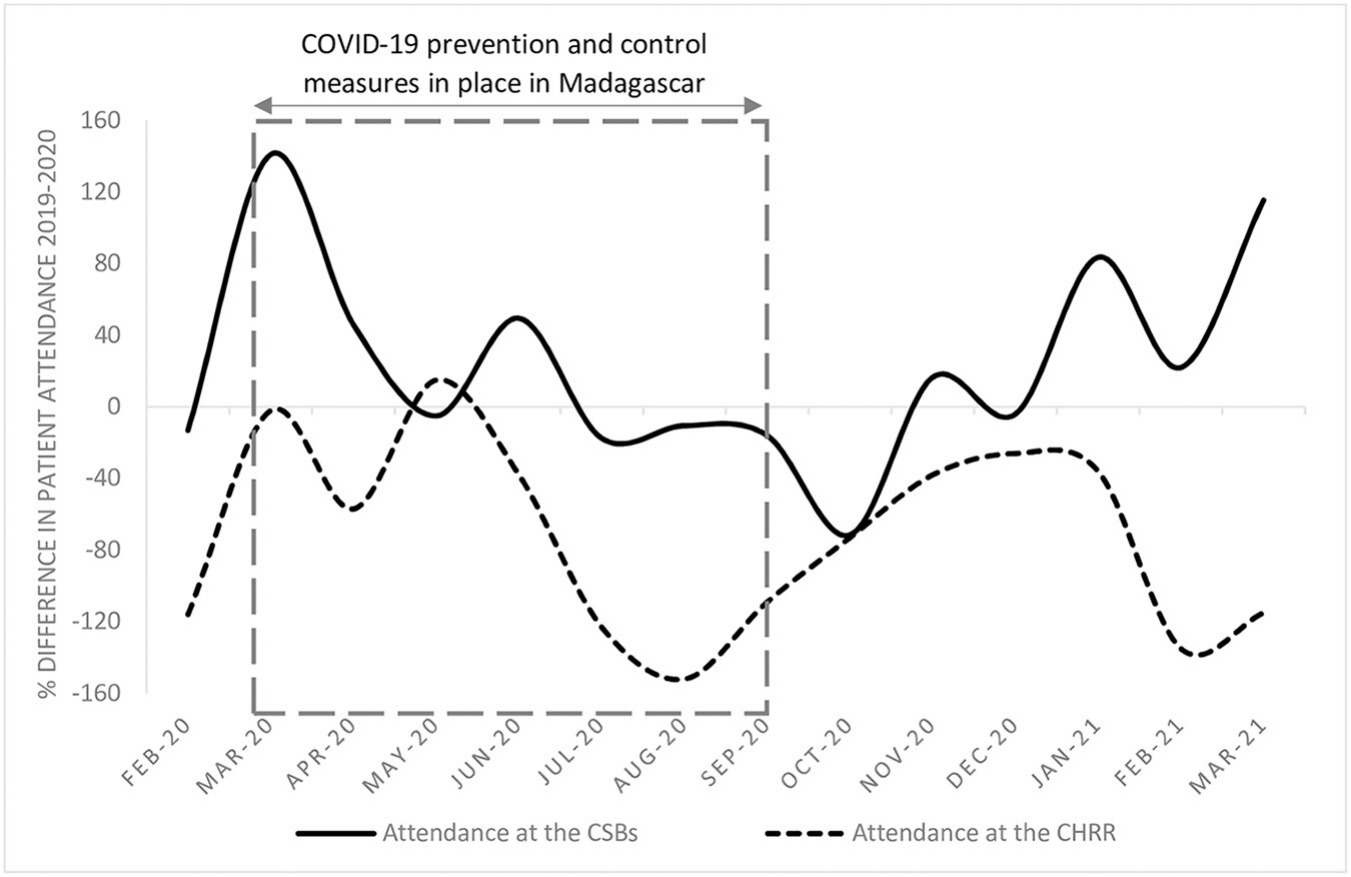
Difference in the number of consultations that took place in CSBs (median) and the CHRR (total number) in Amoron’i Mania from February 2020 to February 2021 compared to the same period the previous year.

There are considerable differences however in attendance at CSBs—primary health facilities serving smaller communities—and attendance at the Centre Hospitalier de Référence Régionale (CHRR)—the regional hospital and designated COVID-19 treatment centre. Fig 1 presents the difference in the number of consultations that took place per month from February 2020 to February 2021 at the 13 included CSBs (median) and at the CHRR compared to the same period the previous year.

While restrictions on travel were in place in Madagascar between March and September 2020, there was an overall decrease in the number of consultations at both the CSBs and CHRR. However, during the entire pandemic period, there was an 11% increase in attendance at CSBs and a 27% decrease in attendance at the CHRR compared to the same period in the preceding year (Table 1).

**Table 1:** Number of consultations taking place in CSBs and at the CHRR before and during the pandemic period.

	Feb 2019–Jan 2020	Feb 2020–Jan 2021	% difference
**CHRR**	2801	2046	-27%
**CSB**	40839	45294	11%
**Total**	43640	47340	8%

### The impact of COVID-19 on plague case notification

**Fig 2:**
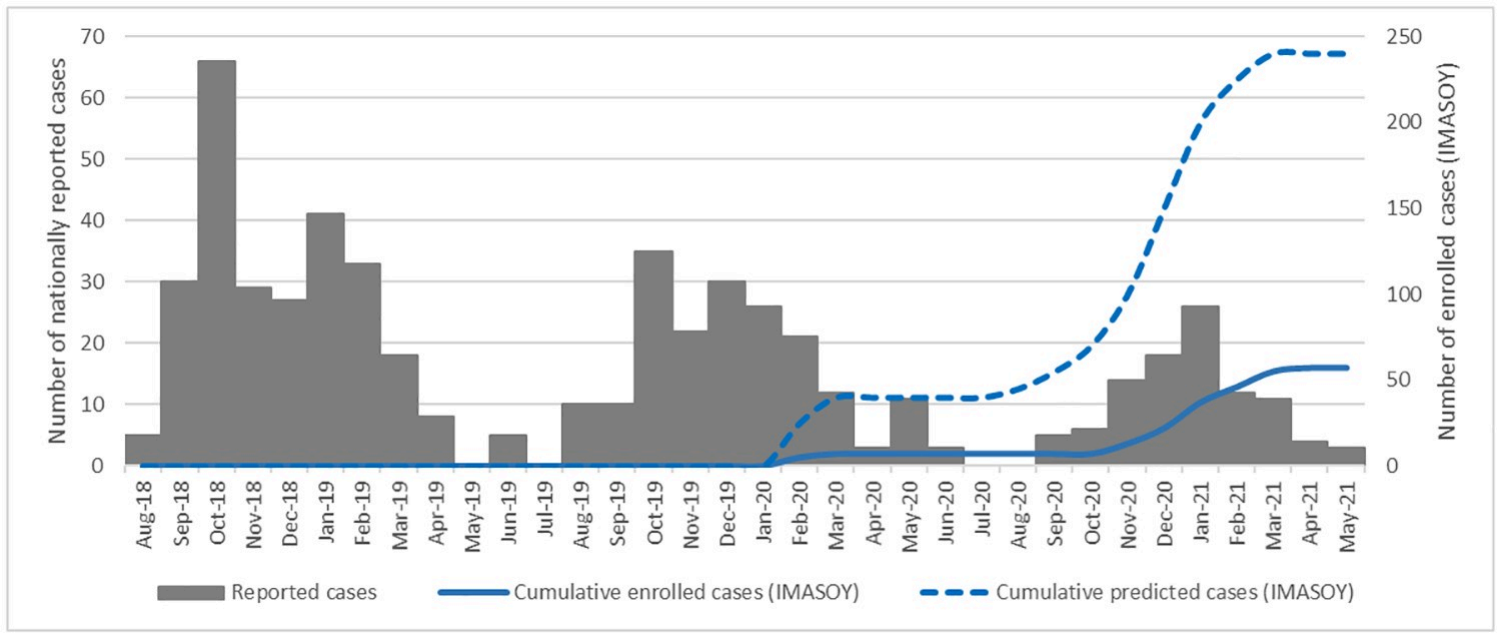
The number of suspected plague cases notified to the national plague programme from August 2018 to May 2021 shown against actual and predicted cumulative enrolment to the IMASOY trial.

We reviewed the national plague surveillance data for three plague transmission seasons (August 2018 to April 2019; August 2019 to April 2020; August 2020 to April 2021) for which an overall decrease in case notification has been observed from 2018 to 2021 (Fig 2).

There were 259 suspected cases of plague notified during the course of the season starting in August 2018, 186 cases notified in 2019 and 105 cases notified in 2020. Between the seasons starting in 2018 and 2019, a 33% decrease in the total number of case notifications per season was observed and between the seasons starting in 2019 and 2020 a 45% decrease was observed between 2019 and 2020. This represents an overall 63% decrease in notified cases of from 2018 to 2020.

This reduction in the total number of notified cases is reflected in the number of districts reporting suspected cases of plague. During the 2018–2019 plague season, 27 districts notified cases with a median of four reported cases per district; while during the 2019–2020 season, 20 districts reported suspected cases of plague with a median of five notified cases per district and during the 2020–2021 season this figure fell further to 17 reporting districts with a median of two notified cases.

### The impact of COVID-19 on recruitment to the IMASOY trial

Between February 2020 and April 2021, a total of 95 cases of confirmed and probable plague were reported to the Central Plague Laboratory; the districts participating in the trial contributed 42 (44%) of the total national cases. Despite the many challenges posed by the COVID-19 pandemic, the IMASOY trial was able to include 25 confirmed cases (60% of the confirmed/probable cases in the participating districts or 31% of the national total (Table 2)).

**Table 2:** Number (%) of confirmed cases of plague reported to the Central Plague Laboratory and number (%) of confirmed cases of plague included in the IMASOY trial (February 2020—April 2021).

	N confirmed and probable cases reported to the Central Plague Laboratory	N (%) confirmed cases included in the IMASOY trial
**Total**	42	25 (60%)
**Ambositra**	25	19 (76%)
**Manandriana**	13	6 (46%)
**Moramanga**	4	0 (0%)
**Tsiroanomandidy**	0	0 (0%)

## Discussion

An indirect link can be drawn between the decrease in notifications of plague cases and the COVID-19 pandemic. The reduction in consultations observed at health centres—which could be attributed to either the restrictions that were placed on domestic travel or fear of contracting COVID-19 at a health centre [15]—may have led patients with suspected bubonic plague to seek care from alternative sources, for example by self-medicating or from traditional healers, [16] meaning that some suspected cases may not have been notified (these cases will be missing from the national data). The same limitations to accessing healthcare were seen during the Ebola (2014–2016) and SARS (2002–2004) epidemics, [17,18] indicating that changes in patients’ health-seeking behaviours is not unexpected.

This interpretation is supported by two findings. First, the overall dip in all-cause consultations during the months of lockdown. Second, the opposite trends at peripheral health centres and the referral hospital: despite its role as a COVID-19 treatment facility, the CHRR observed a 27% decrease in consultations—implying that fewer people travelled to access healthcare during the pandemic, particularly while restrictions were in place.

### The impact of COVID-19 prevention measures on the IMASOY trial

Based on historical national surveillance data, the trial team anticipated randomising approximately 65 confirmed cases per season. In the 14 months since activated included in this review period, the trial enrolled a total of 25 confirmed cases (38% of the initial projection). Whilst multiple recruitment seasons were planned to reach the target sample size, under the current recruitment rate additional seasons will be required, which may bring financial and logistical implications.

The installation of COVID-19 prevention measures has been critical to control the spread of the virus at a national and international level. While a necessary public health measure, restrictions on business activities, travel and gatherings have had an unavoidable impact on the IMASOY trial. In the months between each plague transmission season, the IMASOY trial has a planned recruitment hiatus and the majority of the trial team who are deployed to the participating centres are recalled to Institut Pasteur Madagascar (IPM) in Antananarivo. During this break between April and July 2020, the trial team planned to scale-up the trial to involve more districts and more sites, which involved hiring new staff, conducting training and purchasing more equipment. However, the recruitment of new staff was delayed due to restrictions placed on in-person meetings and business activities. The purchase of trial equipment was also slowed by reduced business activities of suppliers and lengthy import processes for goods purchased abroad. Restrictions on domestic travel and gatherings also resulted in the suspension of all field missions out of Antananarivo, which prevented the timely redeployment of the IMASOY trial team to the participating districts, delaying training and patient enrolment by three months.

Despite these many challenges, the IMASOY trial recruited a representative sample of plague cases—over two-thirds of the those occurring in the participating districts and over one-third of the country cases.

### Mitigation strategy of the IMASOY trial

To mitigate the effects of the pandemic on the IMASOY trial, the trial team have explored several strategies.

The first peak of COVID-19 cases in Madagascar (July 2020) coincided with preparations to restart recruitment for the trial’s second transmission season. In order to support clinicians based in the participating health centres with research activities, when the trial eventually recommenced in November 2020, the number of study technicians deployed to the districts was increased and one technician was allocated to support each site. This allowed patient identification to continue unimpeded and ensure that patient care and data collection were maintained at high, GCP-compliant standards.

Ahead of the recommencing recruitment for the third transmission season in August 2021, the trial team re-evaluated the sites participating in the trial to maximise recruitment potential. Overall, the number of participating districts and sites increased from two districts and 16 sites in 2020 to four districts and 37 sites during 2020–2021 and to ten districts and 39 sites for 2021–2022. The trial was expanded to new locations in Madagascar on the basis of historic case notification. Sites that had not included patients during the previous two transmission seasons were closed in order target the trial’s resources to new locations with higher recruitment potential.

The trial team also plan to submit an extension to the study in 2022 once the effects of the pandemic become clearer. The transmission season starting in August 2021 will be the first undisrupted recruitment season for the trial and will therefore act as an important indicator of the trial’s recruitment potential in subsequent years.

Finally, pausing the trial until COVID-19 transmission stabilises and daily activities return to a new “normal” is another possible option. However, there is an urgent need to identify optimal treatment regimens for bubonic plague and other neglected and re-emerging infections affecting the most vulnerable populations which has not diminished with the arrival of the pandemic. Pausing research efforts risks the delay of these critical results and further neglect of already neglected diseases.

### Reasons for the decrease in plague notification

While there may be an indirect link between the COVID-19 pandemic and a decrease in plague notifications, reduced plague case numbers are likely to derive from multiple factors.

Climate, in particular intense El Niño Southern Oscillation (ENSO) events, is one of the primary drivers behind the prevalence of plague in the environment, which thrives in warm, humid conditions. [19] The 2020–2021 plague transmission season coincided with an La Niña event, which brought cooler, dryer weather in November-December 2020 [20]—coinciding with the typical peak of the plague transmission season in Madagascar.

Plague outbreaks in Madagascar also appear to exist in 4–5-year cycles. [21] However, in hotspot districts, which regularly report cases of plague, cycles appear to be longer—much like those reported in Himachal Pradesh, India which occur in 10–15-year cycles [22]. The climatic influence of ENSO on the environmental cycle of plague in Madagascar, coupled with the periodicity of plague may explain some of the reduction in case numbers reported to the national plague programme. It is possible that the downward trend in case numbers is a result of a natural disease cycle influenced by climate, alongside other ecological factors.

Substantial prevention and control efforts have also been made in some hotspot districts, which combined typically report above 30% of all notified plague cases and significantly influence the plague situation in Madagascar. For example, Ankazobe district, which has historically reported a high number of suspected cases, has put in place prevention measures, such as deinsectisation, rat captures, education and communication, and rapid intervention in case of outbreak alert, that have likely contributed to the decline in case notification. The district reported just two cases in the 2020–2021 plague season.

Restrictions on gatherings imposed by the Malagasy government in response to the pandemic may also have limited human-to-human transmission. In particular, the *Famadihana*, a traditional exhumation practice, is considered to be a significant source of transmission of (bubonic) plague, following which small outbreaks of pneumonic plague have historically been observed. [23]

This trend is not unique to plague. Other neglected and re-emerging infectious diseases with similar transmission patterns have also seen a decline in case numbers in 2021. Cases of Lassa fever in Nigeria, for example, have substantially decreased in 2021 compared to numbers reported in previous years. During the peak of the transmission season (January—April) from 2017–2020, there has been a year-on-year increase in cases of Lassa fever (2017: 164; 2018: 420; 2019: 554; 2020: 991). [24–27] Between January and April 2021 however, only 272 cases were reported. [24,25,28] The decrease in cases of Lassa fever has equally impacted several studies taking place in Nigeria, for which recruitment has slowed considerably.

While the anomalous case numbers reported for neglected and re-emerging infections during the pandemic may derive from multiple sources—one of which may be COVID-19—it is unclear how epidemic-prone diseases will manifest in the years to come while co-existing with COVID-19 or any other new epidemic. The recent decreases in the numbers of confirmed cases of plague and Lassa fever may not be sustained, so we must be prepared for case numbers to rebound and fluctuate. Importantly, the link between many NTDs and climate must not be overlooked. [29,30] Transmission patterns, geographical reach and seasonality of many NTDs is likely to become more unpredictable as climate change increasingly influences how this group of diseases affects human populations. [31]

## Conclusion

The COVID-19 pandemic has had a significant impact on clinical research for neglected and re-emerging infectious diseases. The IMASOY trial is one example of a trial that has suffered unavoidable delays and recruitment challenges that can be traced back to the pandemic. Strategies to mitigate the effect of overburdened healthcare systems have been critical in ensuring that, even with a downward trend in plague case notification at a national level, recruitment and trial activities can be maintained.

While it is possible that the COVID-19 pandemic coincided with an expected decline in the natural disease cycle of plague, it is important to note that the reduction in reporting of infectious diseases during the pandemic does not necessarily mean that there are fewer cases at the community level or that this trend will continue. Resurgence is possible—particularly when patients once again feel more confident to return to health centres. Equally we do not know if concentrating the limited resources of the health system on containing COVID-19 might have also eroded capacity to deploy interventions against plague; the next seasons will tell us. This year therefore should not be viewed in isolation or taken as a marker of things to come. Evidence of plague’s close correlation with ENSO and its cyclical nature should indicate that an increase in cases may be seen within the next few years.

Overall, COVID-19 outbreaks are unlikely to dissipate in the near future. [32] For the next few years while health systems and public trust in healthcare adjust, stakeholders in clinical research must focus on preparedness to continue research in parallel with further upticks in COVID-19 transmission. Strategies, such as those implemented by the IMASOY trial, are examples of ways trials for neglected and re-emerging infections can adapt to this new research environment. As no robust clinical trials have been completed to definitively demonstrate an optimal treatment regimen for bubonic plague, [33,34]—and for many other neglected diseases—it is vitally important that research efforts continue.

## Supporting information

S1 Data IMASOY inclusion data. (CSV)

S2 Data All-cause consultation data. (XLSX)

S3 Data Plague notification data. (XLSX)

S1 Fig Trends in all-cause consultations per site. (TIF)
