# Supplementary figures and images for: The impact of COVID-19 on clinical research for Neglected Tropical Diseases (NTDs): A case study of bubonic plague
Source: PLoS Negl Trop Dis. 2021 Dec 20;15(12):e0010064. doi: 10.1371/journal.pntd.0010064 (PMC8722723; doi:10.1371/journal.pntd.0010064)

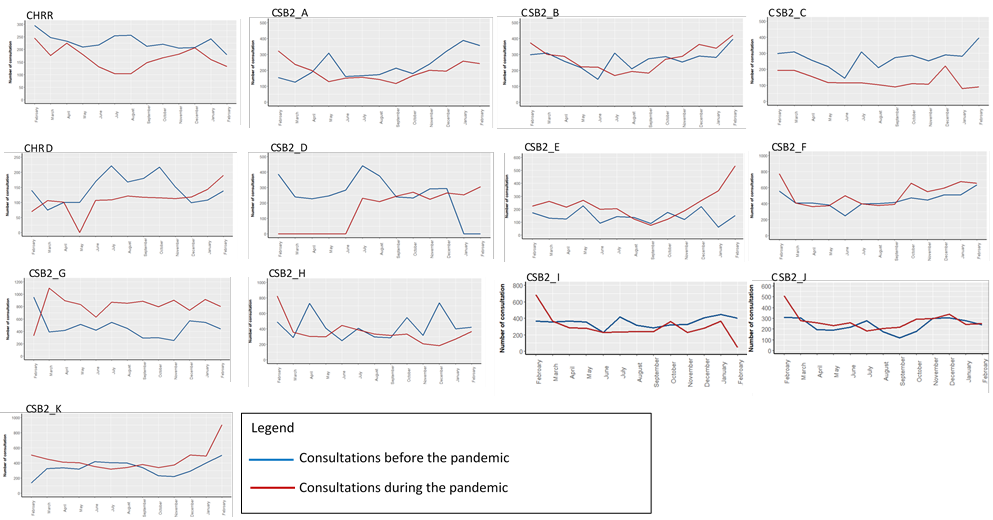

Supplement: S1 Fig — (TIF) [file pntd.0010064.s004.tif]
